# Utilising online eye-tracking to discern the impacts of cultural backgrounds on fake and real news decision-making

**DOI:** 10.3389/fpsyg.2022.999780

**Published:** 2022-12-13

**Authors:** Amanda Brockinton, Sam Hirst, Ruijie Wang, John McAlaney, Shelley Thompson

**Affiliations:** ^1^Department of Psychology, Faculty of Science and Technology, Bournemouth University, Poole, United Kingdom; ^2^Department of Archaeology, Faculty of Social Sciences and Health, Durham University, Durham, United Kingdom; ^3^Bournemouth Business School, Bournemouth University, Poole, United Kingdom

**Keywords:** fake news, online eye-tracking, psychology, culture, cybersecurity

## Abstract

**Introduction:**

Online eye-tracking has been used in this study to assess the impacts of different cultural backgrounds on information discernment. An online platform called RealEye allowed participants to engage in the eye-tracking study from their personal computer webcams, allowing for higher ecological validity and a closer replication of social media interaction.

**Methods:**

The study consisted of two parts with a total of five visuals of social media posts mimicking news posts on Twitter, Instagram, and Facebook. Participants were asked to view examples of real and fake news taken from a news fact-checking website, Snopes, and their eye movements were recorded during the process. Participants were recruited through Prolific and SONA; the total sample size for study 1.1 was 29 participants, and the total for study 1.2 was 25 participants, after removing poor eye-tracking data. A total of five visual images comprising true and false news were shown to the participant, study 1.1 had three examples and study 1.2 had two examples. There were two main cultural backgrounds in focus: participants born in China or the United Kingdom.

**Results:**

Results suggested that participants follow a similar visual pattern of attention to Areas of Interest (AOIs) on the posts, which leads us to believe that due to the global standardisation of popular social media platforms, a bias might have occurred during information discernment.

**Discussion:**

It is suggested that regardless of country background, users may have similar eye-tracking results while viewing a social media post because social media platform formats are standardised globally. Further research would recommend looking at language and linguistic traits when seeking differences between country backgrounds during online information discernment.

## Introduction

As technology evolves, so does virtual space and the way that people behave within it as a user. This has led to the creation of the digital citizen; an individual who is exposed to a vast amount of information. It can be difficult to establish the relationship between the use of the internet and individual behaviour. This is partially due to the characteristics of virtual space, such as the anonymity and potential for identify management that it affords. Interdisciplinary approaches are important to understand the impacts of potentially dangerous information on behaviour, offline. Three areas of research are used to explore the study reported in this article. This includes psychology research used to provide frameworks for cultural and behavioural analysis; media and journalism literature used to clarify current understands of what fake news is; and elements of cybersecurity themes to interpret some dangers of fake news in virtual space.

Fake news has been argued to be a by-product of virtual space and may be used in harmful ways to push agendas (Flintham et al., 2018), similar to propaganda (Tandoc et al., 2018). Some types of fake news most referred to when discussing the harm of information in virtual space include misinformation, disinformation, and malinformation—the last is most closely related to propaganda (Wardle, 2018). Propaganda is traditionally associated with information spread based on political motivations, such as World War II propaganda (Guo and Vargo, 2018). Fake news is not used interchangeably with propaganda, but both have similarities such as its use for the deliberate aim to manipulate based on gain, such as ones that are political (Wardle, 2018). Fake news is suggested to differ when it accepts a wider interpretation of information manipulated to harm, meaning that it is not narrowed down to just political agendas but extends to other spheres, such as revenge porn (*ibid*; Whittaker, 2019). There are several dangers to fake news, including both subversive and overt uses of deceptive information to manipulate members of the public, either domestically or internationally (Bradshaw and Howard, 2019). This type of information spread becomes a cybersecurity and cyberwarfare issue, showing how fake news intersects with other disciplines, when propagated to suppress human rights, dissenting opinions, and discredit political opinions (Bradshaw and Howard, 2019). With this in mind, China was chosen for this study as an example of such a regime that uses computational propaganda to exert information control by repressing dissenting opinions both within and outside its population (Bradshaw and Howard, 2019). This includes operating and restricting access to social media platforms, such as Facebook and Twitter and exerting foreign influence over information spread (*ibid*). Research suggests that these types of authoritarian regimes are more likely to have fewer media outlets and adopt a state-sponsored journalistic approach (Whittaker, 2019). Because of this type of regime, China does not have access to social media such as Facebook, Instagram, and Twitter. This is why in this study we chose participants born in China but who currently live in the United Kingdom, where individuals have access to and familiarity using these platforms. The United Kingdom (UK) similarly engages in computational propaganda (Bradshaw and Howard, 2019). Instead of state-controlled media due to an authoritarian regime, there is research to support the existence of organised manipulation campaigns headed by cyber troops who work with political actors, such as government agencies, politicians/political parties, and private contractors (*ibid*). These types of manipulative campaigns are similar to an authoritarian regime in terms of controlling information narratives in social media, political agendas, and ideas.

Varied definitions of fake news exist across the literature. This difficulty is partially due to the complexity of capturing online ecosystems and pinpointing fake news spread in relation to any specific behaviour phenomenon (Wardle, 2017; Vicario et al., 2019; García Lozano et al., 2020). For example, the truth can be subjective based on an individual’s personal context in which their values and beliefs are shaped (Smith et al., 2016). Echo chambers are one of the more common examples of this in virtual space; this is where individuals commonly select information online they consider correct while ignoring any opposing viewpoints (Flintham et al., 2018). A topic propagated through echo chambers on Facebook includes anti-vaccination disinformation. It is considered radicalised behaviour to share anti-vax information, often based on fraudulent research, perpetuating harmful information-spread that vaccinating children is unsafe (Van Raemdonck, 2019; Cinelli et al., 2021).

Currently, there lacks an agreed epistemological definition of truth that spans all areas and disciplines. Because of this, this article has decided to allow journalistic principles to define truth as *information that is based on evidence and facts* (Graves and Wells, 2019).

Vosoughi et al. (2018) study showed that fake news spreads faster and wider than real news. This is shared by other works, and how those who spread fake news engage and align more strongly with its material (Zhou and Zafarani, 2020; Khan et al., 2021). Colliander (2019) showed that comments and actions of other users on social media impact the reactions to and spread of fake news. Echo chambers, filter bubbles, and algorithms may contribute to the spread of fake news. (Baum et al., 2017; Martin, 2017; Sophr, 2017). Culture is also considered because of works like Rampersad and Althiyabi (2020) who found that culture had the most significant impact on the spread of fake news, with age having as a greater influence on the acceptance of fake news in a particular culture.

### Psychology and cybersecurity

Psychological and cybersecurity research are relevant for this study because of their application in understanding communities and individuals in virtual space, both are discussed as overlapping concepts. Cybersecurity focuses on behaviours and decision-making, with the aim to secure systems from exploitation and manipulation, such as social engineering attacks, like phishing emails (Dreibelbis et al., 2018). Literature by Chadwick et al. (2018) uses studies to explore some of these online behavioural phenomena by looking at fake news and its spread through political groupings of users. Their study showed that Conservative (right-wing political spectrum) supporters are more likely to share fake news and less likely to correct other users for inaccurate news, whilst Labour (left-wing political spectrum) supporters are more likely to encounter inaccurate news and correct other users for sharing fake news. This contributes understanding as to why groupings in virtual space behave as they do. For example, work by Chadwick et al. (2018) work suggests that individuals who identify as more left-wing consume information more consciously by ensuring information is supported by evidence. This acts as a starting point to examine what behaviours may result from individualistic traits and personal alignments.

Kahneman et al. (1982) research discusses the dual process theory of unconscious thinking and decision-making, and this has aided in informing much of the cognitive bias research seen today in psychology (Evans, 2016). Heuristics, also known as unconscious decision-making, covers some of these cognitive biases. These decision-making processes are usually relied upon when there is an abundance of information being presented in our immediate world (Baum et al., 2017). When people face large quantities of information, cognitive thinking is reduced and decision-making capacities are diminished (Martin, 2017). According to Keane (2009), this is partially due to how the profusion of information creates confusion during information discernment. Table 1 provides some examples of heuristics that are suggested to exist in virtual space and discussed in the literature. These heuristics are some of the most relevant heuristics by researchers for this study.

**TABLE 1:** Heuristics in virtual space.

Heuristic	Definition
*Truth bias*	This is where others believe that people are telling them the truth, the layout of social media which uses excerpts of information to tell a story may exacerbate this bias (Shu et al., 2018)
*Acquiescence bias (also known as the agreement bias):*	People who come from hierarchical cultures may be more likely to give extreme responses, this is particularly true for collectivistic cultures (to seem agreeable) (Kitayama and Cohen, 2007)
*Homophily*	The bias where humans for bonds with those that are similar to themselves, Aymanns et al. (2017) finds that the friends of users in social networks influence their friends’ beliefs and their stance towards the news (Chadwick et al., 2018)
*Echo chambers*	Beliefs, ideas, and information are amplified or reinforced by the repetition and communication that occurs online (Martin, 2017)
*Filter bubbles*	Describes how the use of algorithms and filters select what information to expose to users based on their online behaviours like location and search history (Martin, 2017)
*Realism heuristic*	Audio and images are treated by users as more realistic interpretations of the real world of everyday experience over text, for example this can give insight as to why social media posts are more convincing when accompanied by images (Vaccari and Chadwick, 2020)
*Illusory truth effect*	General scepticism could play a role in who falls for FN, describes that those that are more gullible, or having a higher reflexive open-mindedness may be more susceptible to believing FN (Pennycook and Rand, 2020)
*Simple source heuristics*	Relates to more intuitive individuals, those who think that FN is less accurate because they are more likely to pay attention to whether or not the story is coming from a trusted source (Pennycook and Rand, 2020)

Utilising psychology is needed to explore behaviours around information-sharing in virtual space. This is to see if certain conditions, like culture, prompt certain biases when discerning fake news. These biases may cause users to spread fake news topics relevant to their values and beliefs. This may overlap with cybersecurity incidents if it impacts technical systems in government organisations and industries.

### Country profiles and cultural dimensions

Cultural dimensions attach decision-making and behaviour in an easy, clear, and communicable way. This helps to identify potential cultural traits, values, or traditions that may influence information-discernment online at the individual level (Rampersad and Althiyabi, 2020). A bicultural study by Ross et al. (2002) included Euro-Canadian Chinese participants and showed that Chinese-born participants who responded to surveys in Chinese had a greater agreement with Chinese viewpoints than participants in the remaining conditions. The language was used to activate different cultural belief systems in bicultural individuals; their recommendations for additional research is to analyse the social contexts in which cultural differences might be amplified or lessened (*ibid*). Past literature has also suggested that Westerners view themselves in unrealistically positive terms so that they appear better, more in control, and embody inaccurately favourable views of themselves (Endo et al., 2000; Ross et al., 2005). This means that someone who is from the West (like the UK) and who participates in self-report studies, like surveys, may correlate to things like higher confidence to discern information online in our study. Another relevant area that may contribute insights into how information is being discerned based on different country backgrounds is the mindsponge work by Vuong and Napier (2015). Mindsponge discusses the re-evaluation and integration of core values when different countries and cultural backgrounds are implanted into another collective environment that differs from an individual’s original contextual background (*ibid*). For example, when an individual immigrant re-locates to another country they are not native to while they strive to maintain their original cultural heritage while adapting to a new culture (Kizgin et al., 2020).

Literature from Hofstede (1980, 2001) and Hall (1989) helps to establish that web design in virtual space is entrenched in cultural contexts. Web design within a country can follow a polychronic or monochronic style, and as such, cultural dimensions can impact web design by reflecting the collective behaviours and values associated with national and country backgrounds (Vyncke and Brengman, 2010; Moura et al., 2016). A polychronic design, popular in China, is characterised by the heavy use of animations, complex menus, and scrollbars on websites which also relates to low values of Uncertainty Avoidance (UA), seen in Table 2 (Capece and Di Pillo, 2021). Monochronic cultures are associated with web designs that focus more on transparent sites; it tells the users what the links are, and what lies behind them, and impose more linear navigation- associated with more Western contexts (*ibid*). This tells us that there are differences in online usability. Meaning that in the case of web design, layouts are catered according to the geographic access of the website, and expectations for usability differ in cross-cultural settings (Broeder and Scherp, 2017; Shi and Xu, 2020).

**TABLE 2:** Hofstede’s cultural dimensions.

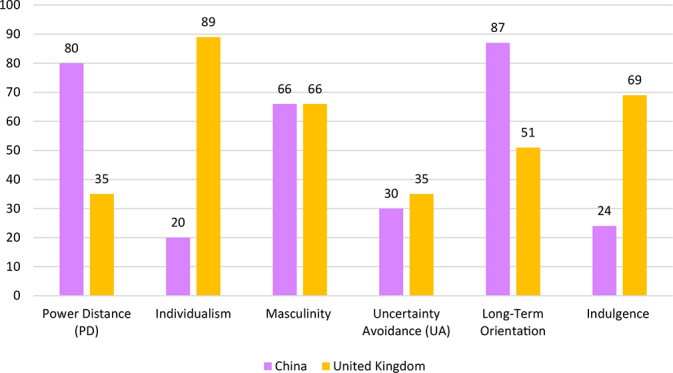

Taken from Hofstede’s Insights, on a scale from 0 to 100 (Insights, 2022). Higher PD = China’s distrust of authorities, knowledge of large power hierarchy difference/inequality between officials and citizens- secrecy heuristic. Lower individualism in China can imply that users are most likely to discern information according to commonly held beliefs and traditions of society, communities, and close connections (family, friends). Higher Individualism can imply a move away from the collective to express identity, this can lead to individuals dissenting from mainstream information falling prey to information cascades such conspiracy theories.

A popular concept that comes up in cross-cultural studies of web design and communication is trust. It is suggested that familiarity with social media platforms like Twitter, Instagram, and Facebook creates a concept of implicit trust. This may not be the case for all users, but as users increasingly experience cultural and technical globalisation, their familiarity with popular social media platforms increases. Trust is suggested to revolve around user expectations, such as social media will behave in a certain way when accessed, that the layout will always be the same and that the navigation of the interface is consistent and predictable. These assumptions have guided what areas would have the most attention, or fixations, in eye-tracking results.

### Eye-tracking

For the disciplines in focus, like psychology, eye-tracking offers the unique opportunity to observe behaviours that contribute to decision-making both consciously and unconsciously. Briefly, this section discusses some advantages of using eye-tracking for our study compared to other data collection techniques. For example, eye movements can indicate subconscious behaviours and decision-making when observing stimuli that may not be self-reported by the participant; self-reports include methods, such as surveys, interviews, and focus groups (Orquin and Holmqvist, 2019). Eye-tracking also allows judgements, decisions, and observations to be collected on the participant over a period of time without interruption to the data collection process (Rahal and Fiedler, 2019) This study utilised both these aspects by recording the eye fixations collated on Areas of Interest (AOIs) and participant’s self-reported qualitative answers about news examples. AOIs are used in this study to indicate pre-defined areas on social media Examples that we thought participants would look at. Self-reported qualitative answers were included to establish if eye-tracking results mirrored participants’ introspection on what they considered traits of real/fake news. Online webcam-based eye-tracking was chosen over surveys and self-report studies due to its more unbiased collection of data (Schmuck et al., 2020). By using a participant’s computer webcam at home versus an eye-tracking lab, it is suggested a better replicate of a real-world environment is provided (Hummel et al., 2017). It is suggested that data collected on underlying cognitive behaviours may have been missed if a participant was relied on to self-report their observations. Online webcam-based eye-tracking was also pursued because of the impact of COVID-19 on lab access to eye-tracking equipment and participants.

It is important to consider traits for visual examples which portray a piece of information as “news” and to reflect how each platform may display news information differently. Social media uses a combination of approaches to communicate stories that include hyperlinks, embedded content, audio, and language (Parikh and Atrey, 2018). These traits aided in mimicking news stories in a virtual environment by replicating posts’ varying visual characteristics found between different social media platforms.

## Materials and methods

There were 34 (Female = 70.6%, age range = 18–42) participants totalled in study 1.1, and 30 (Female = 73.3%, age range = 18–42) participants totalled in study 1.2 after removing poor quality eye-tracking data. Post-experiment, only participants from UK and China were considered bringing the total down to 29 participants (mean age = 22.69, SD =, Female = 21) in study 1.1, and 25 participants (mean age = 22.28, SD =, Female = 19) in study 1.2. All participants had normal or corrected-to-normal vision. A mix of participants including Bournemouth University students was awarded course credits for their participation, and participants were paid £10 through a third-party platform called Prolific. The study was approved by the Bournemouth University ethics board, ethics ID 39420. Qualtrics was used to collect participant consent and demographics. This study’s objectives were to explore differences and similarities in dwell time between participants using online eye-tracking. Dwell time is considered the amount of time a user has looked at a particular area of interest on a screen. Comparisons of dwell time were based on the participant’s country of birth, either born in the UK or China. This comparison was done to see if the participants’ country of birth impacted what AOIs they were dwelling on most. Additional analysis of survey answers aimed to look at discrepancies between participants’ dwell time on AOIs and their self-reported answers. By using both eye-tracking and self-reporting methods, a more holistic representation of how participants are interacting with and process fake news examples is given (Chou et al., 2020).

## Methodology

This study uses a positive approach to analyse quantitative data from experimental data in eye-tracking; this approach is widely used in psychology and is used to explain results from this study (Leahey, 1992; Breen and Darlaston-Jones, 2010). The results are used in a way that supports or dissents from the general hypothesis, and it provides future directions from findings that arose during the study, whether significant or not. A constructivist Grounded Theory approach is also applied to cultural understandings in psychology by allowing for explanations of behavioural nuances like why individuals dissent from the collective. This approach also allows for flexibility in the study’s research outcomes which values methods that explore the social life of individuals and the phenomena surrounding it (Charmaz, 2017).

### Stimuli, materials, and apparatus

A total of five stimuli were created using examples of real and fake news taken from the news debunking website, Snopes. These stimuli imitated the distribution of news across three social media platforms: Twitter, Facebook, and Instagram. A gender-neutral fake user profile was attached to each of the stimuli, this remained consistent across all visual examples. The news stories were chosen to show a combination of both real examples of current news and fake news in circulation in virtual space. The five examples chosen demonstrated wide topic areas that included politics, environment, and current emotive fake news stories. The news stories which the visual examples were based off can be found in Tables 3A,B in this section.

**TABLE 3A:** Eye-tracking visual examples study 1.1.

Example 3
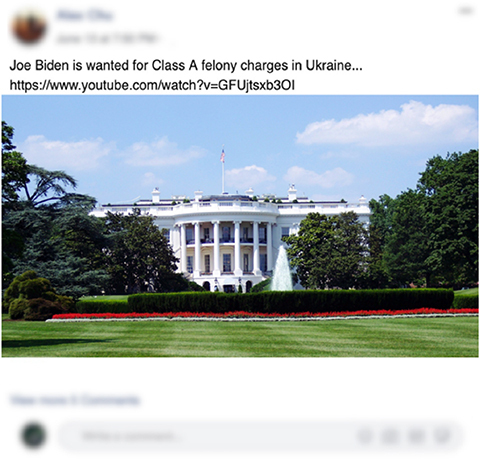

Demonstrates visual Example 3 for study 1.1, two different platforms were used to display news examples in this first study: Facebook and Instagram. These social media examples used a totally fabricated user created and edited by the researchers solely for the research design of this study. Only Example 3 have been demonstrated; Example 1 was based off a news story from Snopes that was proven as false news. The false piece of news information suggested that Coca-cola was associated with the National Socialist German Workers’ Party during the Berlin Olympics of 1936 (MacGuill, 2021). Example 2 was inspired by Readfearn (2021) and discussed The World Heritage Committee’s decision not to put the Great Barrier Reef on the “in danger” list. Example 3 caption inspired by Liles (2021), image taken from Kittredge (2021).

**TABLE 3B:** Eye-tracking visual examples study 1.2.

Example 4	Example 5
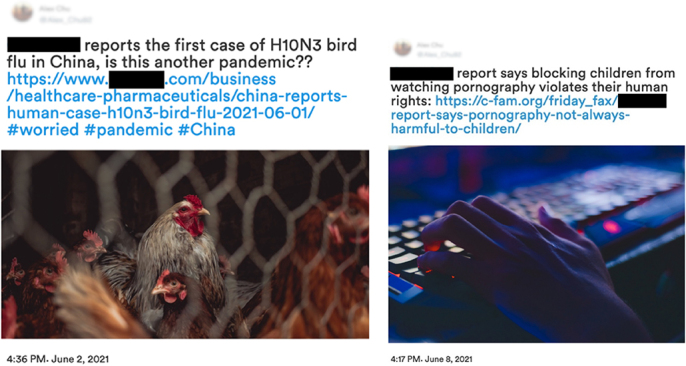	

Demonstrates visual Examples 4–5 for study 1.2, only one platform was used to display news examples which was Twitter. These social media examples used a totally fabricated user created by the researchers solely for the research design of this study. Example 4 caption inspired from Dapcevich (2021), image taken from Kirk (2021). Example 5 caption inspired from Palma (2021), image taken from Kumar (2017).

News cycles are short and usually do not last more than 48 h, so it was assumed that the news stories shared in this study would have already risen and fallen through its natural news cycle (Tan et al., 2016). Other challenges came up when deciding on what news to present. For example, Snopes describes truth as a spectrum, something considered in this study as well, with an example of how truth can vary, shown in Table 4.

**TABLE 4:** Information verification spectrum.

True	This rating signifies that the primary elements of a claim are true and can be demonstrated as so.
Mostly true	This rating signifies that primary elements of a claim can be demonstrated as true, but supplementary details surrounding the claim may be incorrect.
Mixture	This rating signifies that a claim has significant elements that are both true false, and cannot be described by any of the other ratings.
Mostly false	This rating signifies that primary elements of a claim are false and can be demonstrated as so, but there are supporting details surrounding the claim that may be correct.
False	This rating signifies that primary elements of a claim are false and can be demonstrated to be so.

Snopes offers a spectrum of truth classification on news articles they debunk, with the table based off their Fact Check Ratings (Snopes, 2022). For the ease of the study, articles were chosen that were either True or False claims.

The aim for news selection was to demonstrate news items that were either totally false (de-bunked) or totally true (had evidentiary support). We asked participants whether they believe a piece of information to be truthful—based on evidence. This article will focus on the trust of information reflected in “real” classifications and distrust reflected in “fake” classifications. To avoid bias around memory recall, news stories were shared that were not considered viral at the time. Three social media platforms were used to demonstrate news types, this included Facebook, Instagram, and Twitter. The content of the stimuli covered three classifications of news type: environment, politics, and current events. Eye-tracking sought to identify cultural differences during information discernment; listed are assumptions of how researchers thought participants would interact with the stimuli and questions such as:

1. AOIs will be observed differently; those not born in the UK will focus more on the picture while those born in the UK will focus more on the caption/text;
2. Tagging an organisation in a social media post will increase the trust in content;
3. Alex Chu, the made-up user who “shared” news stories, would experience higher distrust from UK users and increased from Chinese users (Maitner et al., 2016);
4. Stimuli that had more interaction would reflect higher trust in content by participants (Colliander, 2019);
5. Hyperlinks would be used to discern the “realness” of the stimuli (Verma et al., 2017);
6. Sensitive topics, such as Examples 1 and 5, (similar to clickbait) would generate higher reactive/emotive responses (Rubin et al., 2015).

After each visual stimulus, a short set of questions asked participants to answer the following:

1. “Is this piece of information real or fake?” (Fake/Real).
2. “How confident are you in your answer?” (Measured on a 4-point Likert scale: Not Confident, A Little Confident, Confident, Very Confident).
3. Based on your previous answer, what characteristic makes this information piece real or fake? (Summarise in a couple of lines).

The stimuli were presented through the online eye-tracking platform RealEye, requiring participants to have access to a computer webcam and the Internet. Due to the technological limitations of RealEye, participants were instructed to complete the experiment without any corrective wear (glasses), but contact lenses were deemed acceptable. The minimum computer requirements to take part in the study as recommended by RealEye were as follows: at least 640 × 480p @ 15 FPS webcam; Google Chrome or Microsoft Edge (10); Windows 7/10 or MacOS X; at least 0.5 GB of RAM memory available; and the minimum resolution 1,024 × 600 pixels. All participants were required to meet these recommendations to take part in the study.

### Procedure

Qualtrics was used to collect informed consent to participate in the experiment, demographic information from the participants, and confirm that the participants met the study requirements. If eligible, they were directed to a test experiment containing a single stimulus on RealEye, this determined whether the participant’s computer webcam met the requirements stated within the previous section of this work. Once this was confirmed, the participants were able to progress to the main experiment. The main experiment was divided into two studies, both to increase concentration and to account for the design variability between the differing types of social media investigated. Study 1.1 comprised three stimuli, representing the distribution of news across Instagram and Facebook; whilst study 1.2 comprised two stimuli, both imitating “screenshots” of news from Twitter. The aim was to present both studies in a randomised order to prevent potential order effects, but technical difficulties only allowed for a randomised order for stimuli in study 1.1; study 1.2 had stimuli 4 introduced followed by stimuli 5. For the description of the eye-tracking calibration process in the RealEye platform, please see Table 5.

**TABLE 5:** Eye-tracking calibration steps.

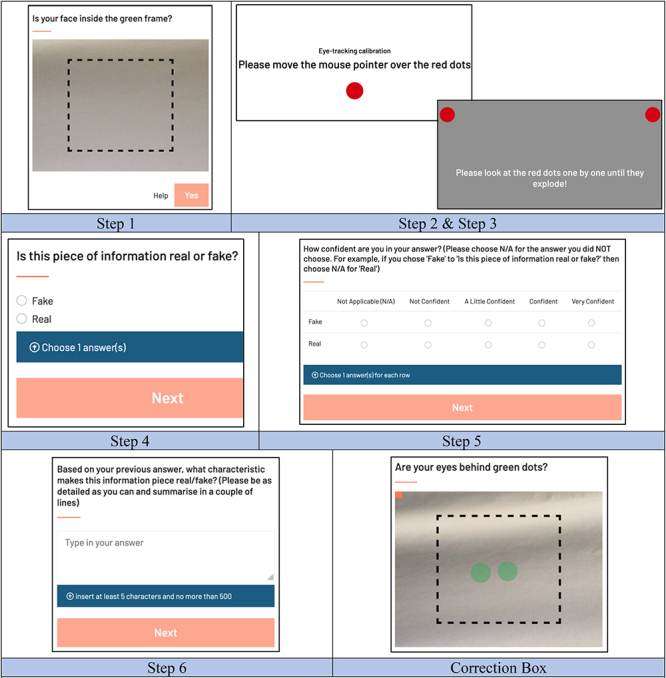

Eye-tracking calibration was achieved through a two-stage process, commencing at the start of each study. First, participants were instructed to frame their head and shoulders in a box (Step 1), then focus on a sequence of 40 calibration points (Step 2), displayed on white, black, and grey backgrounds. This stage required participants to move both their eyes and computer mouse to each point. Participants then completed a further calibration stage (Step 3) involving focus on four points using their eyes. The Stimuli were then presented followed by three (Step 4, 5, and 6) questions about each stimulus. If participants did not pass both calibration stages, they were unable to continue the study. Participants were instructed to keep their head extremely still for the duration of the experiment, with the recommendation that using either a headrest or their arm would boost stability. In the last box, if their head moved at any point, the RealEye program would stop, display a Correction Box, and ask the participant to complete a shorter version of the original calibration process.

Across both studies, participants were exposed to each stimulus for the 20s. This time frame aimed to ensure that participants were able to fully comprehend the stimuli before being asked the survey questions. Before starting the experiment, participants were made aware that they would be assessing the truthfulness of each stimulus; however, the label of what news story types were Fake/Real were not disclosed to the participants. Upon completion of study 1.2, participants were directed to a short de-brief message in RealEye that they were exposed to both real and fake news.

### Data handling

The stimuli within study 1.1 were each allocated five Areas of Interest (AOIs): AOI 1—caption (text), AOI 2—comments, AOI 3—likes, AOI 4—picture (visual), and AOI 5—username. The stimuli within study 1.2 were each allocated four AOIs: AOI 1—caption (text), AOI 2—likes, AOI 3—picture (visual), and AOI 4—username. AOIs were established for each stimulus using RealEye software before being exposed to participants but differed across Study 1.1 and Study 1.2, this was to account for the differences in social media layouts between Instagram, Facebook, and Twitter. Participants were not aware of the AOIs, only the research team had access to what AOI corresponded to what area on the Examples. Data on how participants interacted with AOIs were only available after users had finished the study. These AOIs were developed before the study to indicate the main regions of the social media posts, providing a broad view of the characteristics each stimulus possesses. AOIs were pre-determined by the researcher and are as follows, Examples 1–3: Caption, Comments, Likes, Picture, Username; Examples 4 and 5: Caption, Likes, Picture, Username. A series of analyses were also conducted on the resulting eye-tracking data from both studies, this covered just one eye movement metric: dwell time. Dwell time is a metric of the total time spent looking at a particular AOI, wherein, a higher proportion of dwell time equates to a higher level of visual attention.

### Analysis protocol

Two separate analyses were conducted for each eye movement metric in each study, one investigating the effect of the participant’s opinion on the truthfulness of the news story, and another investigating the effect of each type of social media platform. RealEye allowed for a wide variety of data around eye movements to be collected, due to time and word constraints in exploring all types of eye movements, dwell time is considered the main focus of analysis in this article. Study 1.1 comprised of three visual examples with study 1.2 comprised of two visual examples, there was a fixed exposure time of 20 s for each visual.

First, we wanted to see if there were any differences in dwell time in any of the AOIs between country backgrounds. Second, the country of birth was applied to AOIs and participant’s information discernment of news examples (Fake/Real). This explored potential differences between country backgrounds when it came to information discernment using visual examples of Fake/Real news. Additional insights are then provided to explain how cultural backgrounds are applied to results. Another avenue included in the analysis explores if dwell time for AOIs between examples within studies 1.1 and 1.2 had any differences and/or similarities; this is considered within-stimulus comparisons (Orquin and Holmqvist, 2019). Additional analysis also includes a discussion of the participant’s general discernment of Fake/Real news. Overall, these variables were classified as the most important areas of analysis for this which covers implications of country contexts on information discernment and general trends of Fake/Real news during information discernment of examples.

## Results

Initial tests were explored in Table 6 as crosstabulations to provide general assumptions of how the data might behave and Tables 7A,B describes the crosstabulations of significance tests undertaken. Following the tables, are the significance of dwell times from Study 1.1 and 1.2, the visual Examples of these groups can be found in Tables 3A,B. The significance of the areas of initial exploration surrounding the country of birth is demonstrated to stay aligned with the project’s scope and research outcomes. Any area not explored in this study is recommended in future work.

**TABLE 6:** Crosstabulations of initial assumptions.

Crosstabulations		Assumptions	Observations
Qualitative answer	Opinion news type	An unknown user would reflect skepticism in qualitative answers, and this would lead to lower trust and confidence in information, resulting in more “fake” classifications of news pieces.	“Previous beliefs” was the main qualitative answer participants used to inform labelling visuals as “fake”. For “real” responses, hyperlinks were used for the majority of these two crosstabulations during information discernment.
Confidence scale			Shows that 84.2% are “very confident” in their answer when labelling a piece of information as “fake”; responses for “real” pieces of information mainly fell into the “not confident” part of the confidence scale. This is interesting in that most participants felt not confident discerning a piece of news as real, but were more confident labelling pieces of information as fake.
Country of birth	Confidence scale	Participants born in the UK would be more confident in their news discernment (higher percentage of Confident/Very Confident).	U.K. participants overall answered more “confident” answers in the confidence scale, those from china were mainly grouped by “little confident” confidence scale answers.
	Qualitative answer	Qualitative answers that used hyperlinks to discern between real/fake news would rate higher on the confidence scale.	Between the two crosstabulations, “hyperlinks” are utilised the most by Chinese participants for information discernment, whilst U.K. participants use mainly visual characteristics.
	Opinion new type	Participants born in China would use more visual cues (image, likes) to discern information; participants born in U.K. would use more text to discern information.	Overall, between crosstabulations, participants from China ended up discerning more information as “real” while those from the U.K. had rated more as “fake”.
Qualitative answer	Confidence scale	Participants born in the UK would be more confident in their news discernment (higher percentage of confident/very confident). Qualitative answers that used hyperlinks to discern between real/fake news would rate higher on the confidence scale.	Seen from the crosstabulations, “previous beliefs” had a higher percentage of “confident” answers in qualitative answers and accounted for nearly a third of answers on the confidence scale. “Hyperlinks” came in second influencing “confidence” of qualitative answers. The second highest qualitative used “visual characteristics” to influence “little confidence” on the confidence scale. It seems as if using mainly visual characteristics are not robust enough from a user perspective to effectively inform a piece of news as “real” or “fake”.

**TABLE 7A:** Crosstabulations.

Crosstabulations**		Assumptions
AOIs*	Stimuli display order	Is there significance associated with what AOIs are being viewed and what the display order of news examples is?
	Country of birth Country spent majority of life in	Are there trends according to a participant’s country of birth and country spent majority of life in when looking at AOIs?
	Opinion news type	Is there a trend in AOIs when it comes to real/fake news responses?
Confidence scale	Opinion news type	Are there trends in confidence scales around real/fake responses?
	Country of birth Country spent majority of life in	Are there trends according to a participant’s country of birth and country spent majority of life in and their confidence in their real/fake responses?
	Qualitative answer	Was the confidence scale associated with certain types of qualitative answers?
	Social media type	Were there trends in types of confidence around certain types of social media?
Country of birth	Qualitative answer Opinion news type	Are there trends in country of birth and qualitative answers? Can these qualitative answers align with any specific cultural dimensions seen between UK and China?
Country spent majority of life in		Are real/fake news responses impacted by country spent majority of life in or country of birth?

*This includes the following AOIs, AOI Comment (only in study 1.1); AOI Likes, AOI Picture; AOI Username, and AOI Caption.

**Crosstabulations used Monte Carlo based on 1,000,000 trials and a 99% confidence interval for the *p* value.

**TABLE 7B:** Total dwell time mixed anova results for study 1.1 and 1.2.

Study 1.1: Country of birth x Opinion news type (Real/Fake) x AOI	*F*	*p*	ηp^2^
AOI	(2.75, 74.29) = 55.07	*p* < 0.001***	0.671
Opinion x AOI	(2.15, 57.97) = 10.00	*p* < 0.001***	0.270
Opinion	(1, 27) = 0.63	*p* = 0.433	
Country of birth	(1, 27) = 0.37	*p* = 0.548	
Opinion x Country of birth	(1, 27) = 1.55	*p* = 0.223	
AOI x Country of birth	(2.75, 74.29) = 295	*p* = 0.882	
Country of birth x Opinion news type x AOI	(2.15, 57.97) = 1.13	*p* = 0.332	
**Study 1.2A: Example x AOI x Country of birth**			
Example	(1, 23) = 5.76	*p* = 0.025**	0.200
AOI	(1.65, 38.04) = 18.48	*p* = 0.001***	0.340
Example x AOI	(1.94, 44.59) = 3.53	*p* = 0.039**	0.133
Country of birth	(1,23) = 0.30	*p* = 0.564	
Example x Country of birth	(1, 23) = 0.78	*p* = 0.384	
AOI x Country of birth	(1.65, 38.04) = 1.58	*p* = 0.221	
Example x AOI x Country of birth	(1.94, 44.59) = 0.09	*p* = 0.911	
**Study 1.2B: Country of birth x Opinion news type (Real/Fake) x AOI**			
AOI	(1.59, 36.51) = 14.54	*p* < 0.001**	0.387
Opinion	(1, 23) = 0.001	*p* = 0.993	
Country of birth	(1, 23) = 1.59	*p* = 0.220	
Opinion x AOI	(1.83, 42.02) = 1.51	*p* = 0.232	
Opinion x Country of birth	(1, 23) = 2.22	*p* = 0.150	
Country of birth x Opinion news type x AOI	(1.83, 42.02) = 1.53	*p* = 0.230	

**Significant at *p* < 0.05.

*** Significant at *p* < 0.001.

Table 7B Study 1.1 shows the results of a 2 (Country of Birth: China and UK) × 2 (Opinion News Type: Fake/Real) × 5 (AOI: Caption, Likes, Picture, Username, and Comments) mixed ANOVA conducted on proportion of dwell time to stimuli for study 1.1. This analysis was performed to examine whether dwell time differed between the country of birth, AOIs, and Fake/Real news discernment in study 1.1. Results showed that country of birth [*F* (1, 27) = 0.37, *p* = 0.548] and opinion [*F* (1, 27) = 0.63, *p* = 0.433] had no effect, similar to all remaining effects. There was a significant effect of AOI [*F* (2.75, 74.29) = 55.07, *p* < 0.001, ηp^2^ = 0.671] and the interaction between Opinion*AOI [*F* (2.15, 57.97) = 10.00, *p* < 0.001, ηp^2^ = 0.270]. This means that participants were dwelling on caption, likes, picture, username, and comments differently, demonstrated in Table 8B. AOI 4 (picture) was consistently viewed differently, it can be drawn that this was the most important area dwelled on in study 1.1. Pairwise comparisons for Opinion News Type and AOI show that AOI 4 had significantly (*p* < 0.001) more dwell time compared to all other AOIs for answers associated with participant’s news discernment as “Fake” in study 1.1. For participants who answered labelled news pieces as “Fake” for study 1.1, pairwise comparisons show that AOI 2 received significantly more dwell time compared to AOI 5 (*p* < 0.05). For groups that classified news in this study as “Real,” pairwise comparisons shows that all other AOIs compared to AOI 5 had significantly (*p* < 0.05) more dwell time. This means that AOI 5 had the least dwell time compared to all other AOIs for participant answers that classified a piece of information as “Real.” Pairwise comparisons also show that for participants who labelled news pieces as “Real,” AOI 4 had significantly (*p* < 0.001) more dwell time than AOI 3; AOI 1 had significantly more dwell time than AOI 3 (*p* < 0.005).

**TABLE 8:** **(A)** Descriptive statistics for study 1.1*. **(B)** Descriptive statistics for study 1.2**.

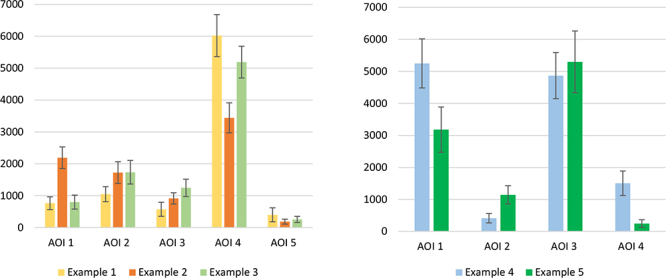

**(A)** Demonstrates the Mean (M) and S.E. of Examples from study 1.1 of AOIs. Example 1: ***AOI 1*** S.E. = 201.007, *M* = 762.878; ***AOI 2*** S.E. = 236.023, *M* = 1048.361, ***AOI 3*** S.E. = 218.562, *M* = 574.003; ***AOI 4*** S.E. = 659.626, *M* = 6021.847; ***AOI 5*** S.E. = 220.244, *M* = 400.767. Example 2: ***AOI 1*** S.E. = 339.739, *M* = 2191.217; ***AOI 2*** S.E. = 341.042, *M* = 1726.089; ***AOI 3*** S.E. = 177.47, *M* = 915.589; ***AOI 4*** S.E. = 470.107, *M* = 3440.917; ***AOI 5*** S.E. = 77.738, *M* = 187.675. Example 3: ***AOI 1*** S.E. = 219.767, *M* = 800.481; ***AOI 2*** S.E. = 369.808, *M* = 1735.631; ***AOI 3*** S.E. = 271.717, *M* = 1247.147; ***AOI 4*** S.E. = 497.468, *M* = 5189.8; ***AOI 5*** S.E. = 96.887, *M* = 256.853. *AOI 1 = Caption, AOI 2 = Comments, AOI 3 = Likes, AOI 4 = Picture, AOI 5 = Username. **(B)** Descriptive statistics for study 1.2^**^. Demonstrates the Mean (M) and S.E. of Examples from Study 1.1 of AOIs. Example 4: ***AOI 1*** S.E = 766.041, *M* = 5251.263; ***AOI 2***: S.E. = 146.509, *M* = 414.285; ***AOI 3*** S.E. = 721.711, *M* = 4869.193; ***AOI 4*** S.E. = 382.526, *M* = 1504.947. Example 5: ***AOI 1*** S.E. = 707.826, *M* = 3181.351; ***AOI 2*** S.E. = 286.096, *M* = 1142.855; ***AOI 3*** S.E. = 963.816, *M* = 5299.224; ***AOI 4*** S.E. = 118, *M* = 246.798. ^**^AOI 1 = Caption, AOI 2 = Likes, AOI 3 = Picture, AOI 4 = Username.

Table 7B Study 1.2A shows the results of a 2 (Country of Birth: China and UK) × 2 (Example: 4 and 5) × 4 (AOI: Caption, Likes, Picture, and Username) mixed ANOVA conducted on the proportion of dwell time to the stimuli for study 1.2. This analysis was performed to examine whether dwell time differed among country backgrounds, AOIs, and Examples in study 1.2. Results also showed that country of birth had no effect [*F* (1,23) = 0.30, *p* = 0.564], but a significant effect [*F* (1, 23) = 5.76, p = 0.025, ηp^2^ = 0.200] of Example emerged. This indicates that participants were dwelling on AOIs between examples 4 and 5 differently, with example 4 having a greater proportion of dwell time spent on it. The interaction between Example and AOI also held significance [*F* (1.65, 38.04) = 18.48, *p* < 0.001, ηp^2^ = 0.340]; in Example 4, there were greater proportions of dwell time for AOI 3 (picture) and AOI 1 (caption). There was no significant difference statistically between AOI 1 and AOI 3, this implies that participants were looking at these AOIs together. Pairwise comparisons in study 1.2 for Example 4 show that AOI 1 was viewed significantly (*p* < 0.001) more than AOI 2 and AOI 4; AOI 3 was viewed significantly more (*p* < 0.001) than AOI 2 and significantly (p < 0.05) more than AOI 4. Pairwise comparisons in this study for Example 5 show that AOI 3 had significantly (*p* < 0.005) more dwell time than AOI 2 and AOI 4; and AOI 1 had significantly (*p* < 0.005) more dwell time than AOI 4.

Table 7B Study 1.2B shows the results of a 2 (Country of Birth: China and UK) × 2 (Opinion News Type: Fake/Real) × 4 (AOI: Caption, Likes, Picture, and Username) mixed ANOVA conducted on the proportion of dwell time to the stimuli for study 1.2. This analysis was performed to examine whether dwell time differed between country background, AOIs, and Fake/Real news discernment in study 1.2. The effect of AOI was found to be statistically significant [*F* (1.59, 36.51) = 14.54, *p* < 0.001, ηp^2^ = 0.387], this would suggest that participants viewed AOIs across examples in study 1.2 differently. The effects of Country of birth [*F* (1, 23) = 1.59, *p* = 0.220.] and Opinion [*F* (1, 23) < 0.01, *p* = 0.993] were not significant, this means that some AOIs were viewed more than others, but the other variables (Country of Birth and Opinion News Type) did not statistically influence fixations on AOIs. Further descriptive statistics are given in Tables 8A,B, on standard error (S.E.), which is the standard deviation of the distribution of sample means taken from a population, and Mean of dwell time for each AOI in study 1.1 and 1.2.

## Discussion

The initial aim of the study was to explore whether a participant’s country of birth had an influence on how participants discerned information across the five visual Examples. Differences were measured between studies 1.1 and 1.2 by looking at mixed ANOVA results for effects of country of birth on Examples 1–5, AOIs, and opinions classifying news types as Fake/Real. Results show that overall, country of birth did not have an effect on AOIs or information discernment of Fake/Real news. AOI 4 (picture) and 1 (caption) were dwelled upon the most compared to other areas in study 1.1, with AOI 3 (picture) being dwelled upon similarly across examples in study 1.2. Results suggested that participants viewed AOIs differently based on their opinions classifying news as Fake/Real in study 1.1. This means that for study 1.1, there was a relationship between AOI 4 (picture) and its role in discerning pieces of news in this study as “fake.” For the same results, participants who classified pieces of news as “real” in the same study may have been informed more by their username, likes, and pictures. It is suggested that those who dwelled on more AOIs had the tendency to label a piece of information as “real,” whereas in the case of information discerned as “fake,” qualitative answers had a more intuitive reason or decisions based on previous beliefs correlating to less AOIs. These previous beliefs and what we labelled *intuitive* decision-making (*I know* statements) are used to reflect the influence of biases, background, and immediate contextual groupings on an individual’s decision-making. The lack of impact from the country of birth implies from a broader scope how linguistic backgrounds are suggested as more impactful during information discernment, which we can see happens in other work (Ross et al., 2002).

One of the most interesting interactions within these examples revolved around how the social media tags of official accounts had some participants perceive Example 5, the Twitter UNICEF Example, in study 1.2 as more trustworthy. Qualitative answers from participants in general reflected higher intuitive statements used to influence their information discernment. This suggests that previously held beliefs were more likely to be used to inform their decision-making on Real/Fake pieces over more logical characteristics, such as images and hyperlinks. This result can indicate how individual decision-making seeks out the “truthiness,” how real something looks, in a statement (Flintham et al., 2018). It is suggested that posts seem truer if they engage with characteristics, such as tagging an organisation, increasing the visual perception of evidence-based news. Another implication, based on the mindsponge intuitive framework, of these interactions is that tags could be used more by individuals from differing country backgrounds to discern whether a piece of information is Fake/Real. Organisations outside an individual’s familiar environmental contexts, or core values, may be trusted more while individuals filter their values. This may be because the evaluation of external values (in this instance information) is still taking place.

Statistical significance in study 1.2 on AOIs 3 (picture) and 1 (caption) affirm past literature which discusses that users rely often on headlines and pictures for information discernment, a format which social media is known for Welbers and Opgenhaffen (2019); Pennycook and Rand (2020). The username was not dwelled on as much, even though qualitative answers reflected scepticism in the user being a stranger. With the Username AOI fixated on the least, it suggests that people are interested in the content more than they are interested in who posted the social media post. One explanation considers participants’ qualitative answers, reflecting that social media is not considered an “official” source of news. This has implications around whether people are more likely to believe trusted sources online. For example, it is known that information which is believed and shared in virtual space does not always come from an official news source. And secondly, information that comes from an official news source can be rejected by a user based on unconscious behaviours, such as confirmation bias. Both are characteristics of the post-truth age which is the rejection of expert advice and objective facts in favour of belief systems and opinions that integrate easier into an individual’s reality, such as biases, or trusting peers over experts in social media (Flintham et al., 2018).

There were limitations in this study, the sample size was smaller than aimed for with an imbalance in country backgrounds. An imbalance in country backgrounds limits the ability to represent populations, therefore results are provided as snapshots of collective and individual behaviours. This study was undertaken during the COVID-19 pandemic, restricting access to traditional eye-tracking tools. Challenges arose when deciding what news stories would best represent real and fake news. This included considering questions such as: how “fake” the Examples needed to be; how “fake” news can vary between environmental contexts; and what the definition of “truth” is in our current research. Other challenges included considering how our results compared to other research in this field because definitions of fake news vary across the literature. It was agreed that current pop culture references to fake news examples would not be used, this was to avoid answers based on long-term memory recall and bias that may occur with information discernment and the illusory truth effect (Pennycook et al., 2018). The participants were not told explicitly from the beginning that the study comprised of real and fake news, but this was evident once the participant was asked to label the type of news they were exposed to.

### Future research

Discussed in this section are the implications results have on future research. While reviewing results, much of the eye-tracking data was non-significant, but there may be an explanation to describe some of these phenomena. Globalisation is suggested to have an impact not only on culture, people, language, and traditions but on social media platforms as well. This can be seen through the standardisation of social media platforms, such as Twitter, Facebook, and Instagram. Suggesting that when a social platform is accessed by a user, the user expects it to act and deliver information in a certain, standardised way. The foundational structure of the platform does not change, but the information does. This means that usernames, pictures, captions, and comments are typically found in similar areas on a social media platform; participants may instinctually behave more collectively even from varying country backgrounds while assessing information pieces in this study.

This study showed a lack of significance of country background across individual information discernment of pieces of news as Fake/Real. This suggests that a more linguistic look at qualitative interactions to seek if differences around cultural nuances emerge. Language could be a key factor for activating belief systems in bicultural individuals, this may be especially important to consider for bicultural Asian participants or Chinese and English speakers in this study. Bicultural backgrounds may be more sensitive to language differences because different languages trigger different cultural dimensions; Chinese reflects more collectivist traits and English is more individualistic (Kanagawa et al., 2001). These traits can impact information discernment online. It is suggested that confidence in identifying fake and real news is impacted more by the collective in countries like China; higher ecological validity would replicate interactions of these users by mimicking these instances of situational cues. Studies could focus on platforms that are utilised the most according to unique cultural contexts. For example, exposing Weibo to the UK and Chinese cultural backgrounds; this could increase the opportunities to explore differences. Future work in eye-tracking could also consider linguistic nuances to build off this study to explore if information discernment is similar past a certain education level. Education level should be considered because it may be that past a level of education, similar ways of discerning and critically evaluating information may occur, or there is a higher chance that individuals are already inoculated against mis/dis/malinformation which may both create similar results to what we see in this study.

Overall, eye-tracking was used in this study to examine if the country of birth impacted the participant’s dwell time on AOIs. We also looked at whether discernment of news as Fake/Real was impacted by country of birth and reflected in AOIs while exploring if differences/similarities existed between visuals in each study. Country of birth was not significant, but themes suggested that certain AOIs received more dwell time than others and Fake/Real answers were statistically significantly associated with AOI pictures. It is recommended for future work from this data set to focus on eye-tracking results and participant interactions around Fake/Real news Examples. This avenue of research can delve into why participants were focusing on certain AOIs over others based on their information classifications, revealing more about an individual’s unconscious behaviours during news discernment.

## Data availability statement

The raw data supporting the conclusions of this article will be made available by the authors, without undue reservation.

## Ethics statement

The studies involving human participants were reviewed and approved by The Science, Technology and Health Research Ethics Panel, Bournemouth University. The patients/participants provided their written informed consent to participate in this study.

## Author contributions

AB was the lead author who contributed to the structure, article content, and study. SH contributed to the content on eye-tracking and statistics. RW contributed to the content on online eye-tracking. JM and ST contributed by advising on the research literature and data analysis. All authors contributed edits and approved the submitted version.
