# Corrigendum: Utilising online eye-tracking to discern the impacts of cultural backgrounds on fake and real news decision-making

**DOI:** 10.3389/fpsyg.2023.1140818

**Published:** 2023-02-28

**Authors:** Amanda Brockinton, Sam Hirst, Ruijie Wang, John McAlaney, Shelley Thompson

**Affiliations:** ^1^Department of Psychology, Faculty of Science and Technology, Bournemouth University, Poole, United Kingdom; ^2^Department of Archaeology, Faculty of Social Sciences and Health, Durham University, Durham, United Kingdom; ^3^Bournemouth Business School, Bournemouth University, Poole, United Kingdom

**Keywords:** fake news, online eye-tracking, psychology, culture, cybersecurity

In the published article, there was an error in the Funding statement. The grant number was mistakenly omitted. The correct Funding statement appears below.

This material is based upon work supported by, or in part by, the Army Research Laboratory and the Army Research Office under contract/grant number W911NF-19-0419.

The authors apologize for this error and state that this does not change the scientific conclusions of the article in any way. The original article has been updated.

